# Left Atrial Low-Voltage Extent Predicts the Recurrence of Supraventricular Arrhythmias

**DOI:** 10.3390/jcdd11100334

**Published:** 2024-10-21

**Authors:** Yannick Teumer, Luca Gold, Lyuboslav Katov, Carlo Bothner, Wolfgang Rottbauer, Karolina Weinmann-Emhardt

**Affiliations:** Internal Medicine II, Ulm University Hospital, Albert-Einstein-Allee 23, 89081 Ulm, Germany; yannick.teumer@uniklinik-ulm.de (Y.T.); wolfgang.rottbauer@uniklinik-ulm.de (W.R.)

**Keywords:** left atrial cardiomyopathy, fibrosis, recurrence prediction, supraventricular tachycardia, low voltage, 3D mapping, atrial fibrillation, atrial flutter

## Abstract

The incidence of left atrial (LA) supraventricular arrhythmias is increasing. Even after LA ablation, recurrence of these tachycardias is common. MRI studies show that LA cardiomyopathy is a significant risk factor for recurrence and correlates with low voltage areas detected via 3D electroanatomic mapping (EAM). There are limited data on the impact of low voltage extent detected by EAM on recurrence-free survival. Voltage thresholds defining low voltage vary across different studies. This study aims to investigate the impact of the extent of low voltage areas in the LA on recurrence-free survival and to assess whether defining low voltage areas using thresholds of 0.5, 0.4, or 0.3 mV offers better predictive performance. Patients with atrial arrhythmia who underwent LA EAM at Ulm University Heart Center between September 2018 and September 2022 were included from the ATRIUM registry. ROC analysis determined the voltage threshold for predicting recurrence-free survival. Kaplan–Meier and logistic regression models adjusted for patient variables were used to analyze recurrence-free survival. Of 1089 screened patients, 108 met the inclusion criteria. ROC analysis indicated that a 0.4 mV threshold for low voltage provided the best predictive performance. Logistic regression showed a 1.039-fold increase in recurrence risk per percent increase in LA low voltage area (odds ratio = 1.039, 95% CI 1.014–1.064). Low voltage extent in EAM correlates with 1-year recurrence rate after ablation of left atrial supraventricular arrhythmias. The threshold of 0.4 mV is the most suitable for predicting recurrences of those examined.

## 1. Introduction

The incidence of complex supraventricular arrhythmias is on the rise [1,2,3,4], with many originating from the left atrium. Left atrial (LA) cardiomyopathy is a known predisposing factor for atrial tachycardia (AT) [5,6]. Due to conduction anomalies, LA cardiomyopathy, mainly driven by fibrosis [7,8], favors the development of re-entry tachycardias and the maintenance of atrial fibrillation (AF) [5,9]. Thus, LA cardiomyopathy can be referred to as the substrate for atrial fibrillation and other AT [10].

LA cardiomyopathy can be induced by various factors, including mitral valve pathologies [11], persistent AF [12], and iatrogenic causes such as ablation procedures. These tissue alterations can be detected using late gadolinium enhancement (LGE) sequences with magnetic resonance imaging (MRI) [9,13]. Several studies have identified atrial tissue fibrosis measured by MRI as a key predictor of atrial tachyarrhythmia recurrence following catheter ablation in AF patients [6,10,14,15].

Currently, 3D mapping-guided ablation is considered the gold standard for the treatment of supraventricular arrhythmias such as AF and atypical atrial flutter (LAFL). Thereby, a strong correlation between LGE-MRI enhancement and the low voltage in the EAM has been demonstrated [15,16,17]. As a result, areas of low voltage in the bipolar voltage map can be a surrogate of atrial cardiomyopathy. While cardiac MRI is an additional preprocedural step, LA cardiomyopathy can be localized intra-procedurally by 3D electro-anatomical mapping (EAM) technology [18].

However, few data are currently available on the impact of low voltage detected by 3D mapping on post-interventional recurrence-free survival. Furthermore, different definitions of low voltage in the left atrium can be found in the literature, and the optimal voltage threshold to detect left atrial substrate is unclear [13,17].

The objective of this study was to evaluate the impact of the extent of low voltage areas in the left atrium, as measured by EAM, on the predictive value for post-ablation recurrence-free survival. Additionally, the study aimed to investigate whether defining low voltage areas with a threshold of 0.5, 0.4, or 0.3 mV offers better predictive performance for recurrence-free survival after intervention.

## 2. Materials and Methods

### 2.1. Study Design

In this study, patients with atrial arrhythmia who underwent 3D mapping of the LA at Ulm University Heart Center, Ulm, Germany, between September 2018 and September 2022, were screened. To ensure comparability, only patients treated with a single 3D mapping system (CARTO3 system, Biosense Webster, Irvine, CA, USA) were included. Exclusion criteria included mapping in a rhythm other than sinus rhythm, incomplete LA map anatomy, fewer than 1000 electro-anatomical points, clinical follow-up of less than 6 months, and redo procedures for the same patient during the study period from 2018 to 2022. All patients in the study gave written informed consent prior to 3D mapping and ablation procedure. The study complies with the Declaration of Helsinki and was approved by the local ethics committee of the University of Ulm (reference number: 324/16). The data were collected as part of the ATRIUM registry (German Clinical Trials Register-ID: DRKS00013013).

### 2.2. Mapping Protocol

The workflow for transseptal access to the left atrium has been comprehensively detailed by our group previously [19]. Following the establishment of dual transseptal access, mapping was conducted using a steerable sheath (Vizigo, 8.5F, Biosense Webster, Irvine, CA, USA) in conjunction with a multipolar mapping catheter (Lasso or Pentaray, Biosense Webster, Irvine, CA, USA) and the 3D mapping software (Carto 3, Biosense Webster, Irvine, CA, USA). The Lasso catheter has 20 electrodes, each 1 mm in size, which are arranged with an interelectrode spacing of 2-6-2 mm. The Pentaray catheter used also has 20 electrodes, each 1 mm in size, which are arranged with an interelectrode spacing of 2-6-2 mm. Mapping was performed during sinus rhythm. In cases where patients presented with atrial fibrillation (AF) as the initial rhythm at the start of the procedure, electrical cardioversion was performed to restore sinus rhythm before mapping. Mapping was performed always using tissue proximity filter.

### 2.3. Map Analysis

**Figure 1:**
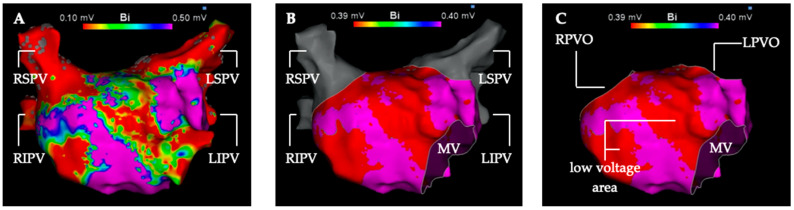
Depiction of the segmentation and measurement process of the left atrial electro-anatomical map into the left atrium and pulmonary veins. High-density 3D voltage-map of the left atrium in anterior-posterior view (**A**) with the default voltage settings (0.1 mV–0.5 mV) and without map editing. (**B**) Voltage setting for detection of low voltage areas <0.4 mV (red color). Left atrial low voltage (substrate) shown in red at the anterior and septal wall. High-voltage > 0.4 mV is depicted in purple color. The mitral valve is cut out and the pulmonary veins (gray color) are separated from the left atrium. (**C**) Left atrial voltage map in the voltage settings as in (**B**). The four pulmonary veins were excised in pairs along their anatomical boundaries. LIPV, left inferior pulmonary vein; LPVO, left pulmonary vein ostium; LSPV left superior pulmonary vein; MV, mitral valve; RIPV, right inferior pulmonary vein; RPVO, right pulmonary vein ostium; RSPV, right superior pulmonary vein.

The low voltage analysis was performed in the first LA map of the procedure generated before ablation therapy. Additional maps of the LA created during the procedure were not included in this analysis. The first step was to segmentate the left atrial map into pulmonary veins (PV) and the LA for volumetric measurement (Figure 1). The second step was to measure the total surface of the LA with cut-out PVs and mitral valve. In a third step, every low voltage area in the LA was measured in detail for three voltage thresholds (0.3 mV, 0.4 mV, 0.5 mV).

### 2.4. Catheter Ablation

In all patients, ablation was performed using a 3D mapping-guided, radiofrequency catheter (Smarttouch SF, 8F, Biosense Webster, Irvine, CA, USA) under deep sedation [20]. Different ablation targets were chosen depending on the type of arrhythmia. For AF without prior pulmonary vein isolation (PVI), PVI was performed as the sole procedure. PVI was confirmed by demonstrating both entry and exit block. In cases of AF recurrence in patients who had already undergone PVI, the first step was to check whether all pulmonary veins remained isolated. If reconnection was detected, focused reisolation of the vein affected was performed. If complete isolation was confirmed at the beginning of the procedure, a substrate modification was performed, followed by non-PV trigger ablation if necessary. For substrate modification, box isolation was performed according to the patient’s individual low voltage localization and extent. For LAFL, substrate-based lines were applied [21]. A bidirectional block was tested over applied lines. For focal atrial tachycardias, focal left atrial ablations were performed.

### 2.5. Clinical Follow-Up and Periprocedural Management

Patients were scheduled in our outpatient clinic for follow-up with 12-lead resting ECG, and 7-day holter ECG at 3, 6, and 12 months after ablation. During a 90-day blanking period, no ablation procedures were performed. Any atrial arrhythmia (AF, AT, AFL) >30 s was considered a recurrence. Antiarrhythmic drugs, except beta blockers, were stopped preprocedural. 

### 2.6. Statistics

Statistical analyses and graph generation were conducted using SPSS Statistics (version 29.0.1.0, IBM, Armonk, NY, USA). Descriptive and inductive statistics were applied based on the level of measurement. A significance level of *p* < 0.05 was established.

Categorical variables were depicted as frequencies and analyzed using either the Chi-square test or Fisher’s exact test, selected as appropriate. Numeric variables were represented as mean ± standard deviation (SD) or as median (interquartile range, IQR), as appropriate. For variables demonstrating normal distribution and homogeneity of variance, inductive testing was performed either with student’s *t*-test or ANOVA (analysis of variance), with normality confirmed via histograms and homogeneity assessed by Levene’s test. In cases where assumptions were not met, the Mann–Whitney U-Test was used.

The receiver operating characteristic (ROC) analysis was conducted to determine the optimal voltage threshold for detecting low voltage, aimed at maximizing the area under the curve (AUC). Survival analysis utilized the Kaplan–Meier method, with group comparisons facilitated by the log-rank test. Based on the LA low voltage extent, patients were categorized into four low voltage stages (stage I < 25%, stage II 25–50%, stage III 50–75%, and stage IV 75–100% LA low voltage areas). The extent of low voltage was defined as the proportion of the surface area in the LA map, with a voltage below the determined low voltage threshold with the highest predictive value in the ROC analysis, relative to the total surface area of the left atrium. Logistic regression was utilized to assess the influence of various factors on the probability of recurrence within the initial year post-ablation. Model validity was verified through the Omnibus test and the Hosmer–Lemeshow adjustment statistic.

### 2.7. Study Endpoints

The first endpoint of our analysis was to determine which voltage threshold (0.3 mV, 0.4 mV, 0.5 mV) for defining low voltage areas in EAM offers the best predictive performance for post-interventional AT/AF recurrence. The second endpoint of this study was to analyze the influence of the LA low voltage extent in EAM and their predictive value on post-ablation AT/AF recurrence.

## 3. Results

### 3.1. Study Population

**Figure 2:**
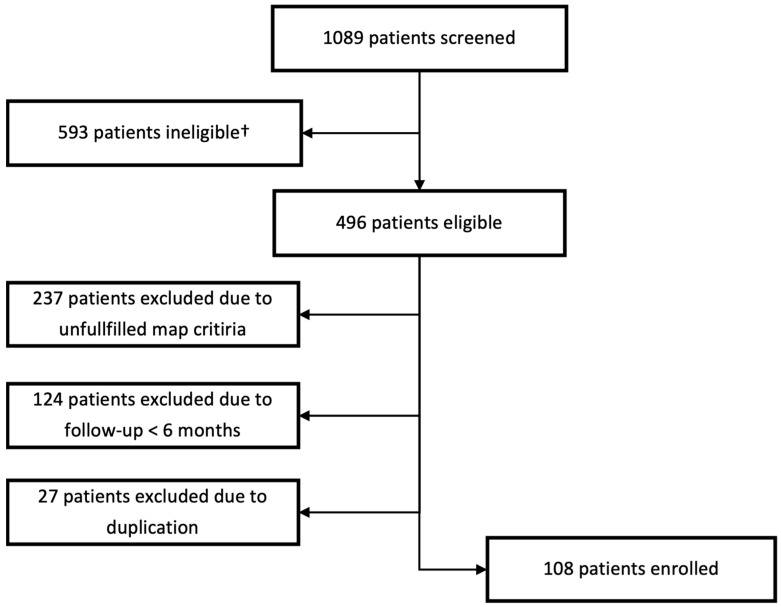
Selection process of the study population. † Patients ineligible due to usage of other 3d mapping system.

In total, 1089 patients were screened from the ATRIUM registry. In the end, 108 patients met all criteria and were included in the study. For further details on the reasons for exclusion, see Figure 2.

### 3.2. Baseline Characteristics

At the time of index ablation, patients had a mean age of 68.8 ± 9.7 years. Of the 108 patients studied, 44 (40.7%) were women and 64 (59.3%) were men. The majority (*n* = 80, 74.0%) had already undergone previous left atrial ablation. There were 53 patients in LA low voltage stage I (49.1%), 32 patients in stage II (29.6%), 16 patients in stage III (14.8%), and 7 patients in stage IV (6.5%).

Age (*p* < 0.001), gender distribution (*p* < 0.001), and left ventricular ejection fraction (LVEF) (*p* = 0.036) differed significantly among the LA low voltage stages. No statistical difference was found between the cohorts for the other baseline characteristics. A comprehensive overview of the baseline characteristics of our final cohort (n = 108) is provided in Table 1.

The analyzed 3D-EAMs had a median (IQR) of 1793 (1254–2488) mapping points. In 96 patients, AF was the indication for the procedure, while in 12 patients another supraventricular arrhythmia was the indication. A total of 120 supraventricular tachycardias were treated in the left or the right atrium. More detailed information regarding the treated arrhythmias and the concomitant ablation therapy is presented in Table 2 and Table 3.

### 3.3. Receiver Operating Characteristic Analysis

**Figure 3:**
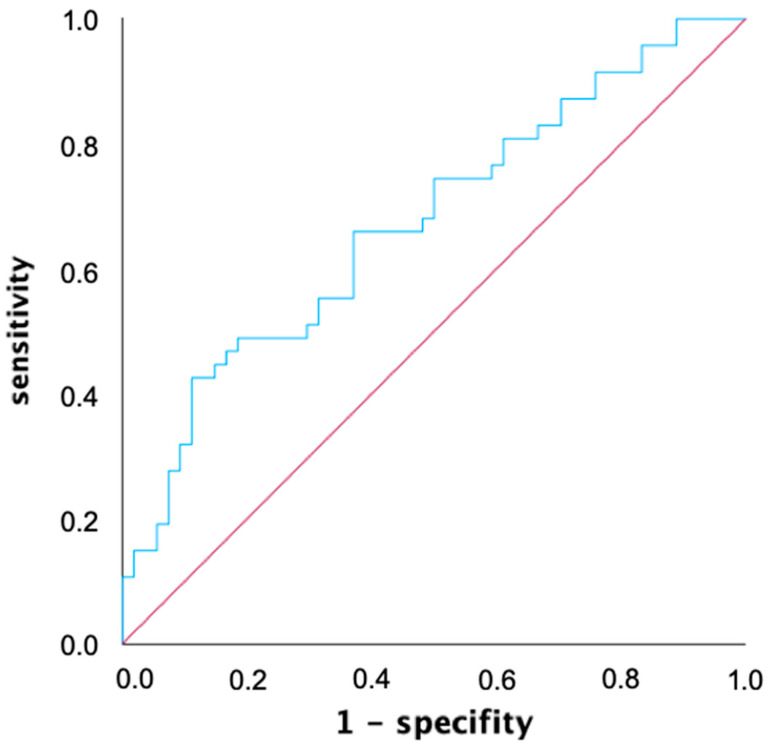
Receiver operating characteristic (ROC) graph of left atrial low voltage extent for predicting atrial tachycardia recurrence within one year after ablation with a voltage threshold of 0.4 mV. ROC analysis allows for the performance of a quantitative diagnostic test to be evaluated by calculating and displaying sensitivity and specificity in a diagram. The area under the curve (AUC) for the voltage threshold of 0.4 mV defining low voltage is 0.675.

An ROC analysis was used to determine a common voltage threshold that provides the highest predictive power for both mapping catheters in predicting atrial tachycardia recurrences within the first year after ablation. The AUC of the ROC curve was determined to be 0.675 for the 0.4 mV threshold, and was slightly better compared to the two other thresholds (AUC 0.3 mV: 0.667, AUC 0.5 mV: 0.674) with simultaneous consideration of both mapping catheters (Figure 3). When considering both mapping catheters separately, the best predictive value for mapping catheter 1 (Lasso, Biosense Webster, USA: AUC 0.3 mV: 0.709, AUC 0.4 mV: 0.711, AUC 0.5 mV: 0.715) was at 0.5 mV, while for mapping catheter 2 (Pentaray, Biosense Webster, USA: AUC 0.3 mV: 0.587, AUC 0.4 mV: 0.615, AUC 0.5 mV: 0.608), it was at 0.4 mV.

### 3.4. Arrhythmia Recurrence Analysis

The mean follow-up time was 437 ± 354 days after the procedure. Seven patients had a follow-up duration less than 12 months. The clinical endpoint of recurrence of any supraventricular tachycardia (including AF and AT) was observed within the first year after ablation in 47 out of 101 patients (46.5%). Looking at the subpopulation of atrial fibrillation patients (n = 96), 42 out of 96 patients (43.8%) had an AF recurrence after 12 months, while 21 out of 96 patients (21.9%) were diagnosed with a non-AF recurrence after 12 months. In the overall population of this study, patients who experienced AT/AF recurrence within one year after ablation had a significantly larger extent of low voltage in the left atrium (LA) compared to those who remained recurrence-free (38.7% ± 26.5% vs. 23.4% ± 20.0%, *p* = 0.001). In the subpopulation of AF patients, those with AT/AF recurrence exhibited a significantly greater extent of low voltage in the left atrium, both numerically and statistically, compared to AF patients without recurrence (36.7% ± 24.6% vs. 23.4% ± 20.3%, *p* = 0.006). In contrast, in the subpopulation of non-AF patients (n = 12), those with AT/AF recurrence showed a numerically greater extent of low voltage in the left atrium, but this difference was not statistically significant (46.9% ± 26.8% vs. 39.2% ± 25.2%, *p* = 0.654).

**Figure 4:**
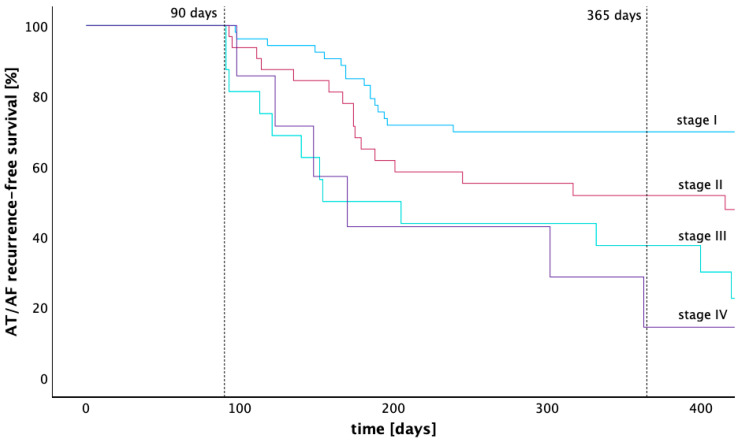
Kaplan–Meier graph of recurrence-free survival across four different stages of low voltage extent. This Kaplan–Meier graph compares the recurrence free survival of four groups with different extent of low voltage in the left atrium after a 90-days blanking period. Stage I is defined as having less than 25% low voltage extent of the left atrial endocardial surface, stage II as 25–50%, stage III as 50–75%, and stage IV as 75–100%.

The rate of freedom from atrial tachycardia 12 months after ablation calculated by the Kaplan–Meier estimator was 69.8% (stage I), 51.7% (stage II), 37.5% (stage III), and 14.3% (stage IV). The Kaplan–Meier graph in Figure 4 illustrates the arrhythmia-free survival of patients across LA low voltage stages I to IV. A pairwise comparison of the Kaplan–Meier estimators for arrhythmia-free survival was performed using the Log-Rank test. Significantly higher arrhythmia-free survival rates were found for stage I compared with stages II, III, and IV (*p* = 0.025, *p* < 0.001 and *p* = 0.003). The arrhythmia-free survival of patients from stage II was also significantly higher compared to stage III (*p* = 0.031), while statistical significance was lacking when compared with stage IV (*p* = 0.129). No significant difference in arrhythmia-free survival was found between stage III and stage IV (*p* = 0.960). After 12 months, 96 patients were treated with beta blockers, 1 patient was treated with flecainide, and 1 patient was treated with amiodarone.

### 3.5. Regression Model

A logistic regression model was performed to estimate the influence of LA volume, LA low voltage extent adjusted for age, sex, BMI, and relevant mitral valve disease on the one-year recurrence risk. The omnibus test for this regression model was as desired significant (*p* = 0.039) and the Hosmer–Lemeshow test was as desired non-significant (*p* = 0.184). The low voltage extent was the only variable showing a significant influence (*p* = 0.002). For every percentage point in LA low voltage extent, we calculated a odds ratio of 1.039 (CI 1.014–1.064) for the recurrence probability of an AT or AF. Table 4 provides an overview of these results.

## 4. Discussion

The main findings of this study are:

- Patients with AT/AF recurrence after 12 months had a significantly higher proportion of low voltage areas in the left atrium compared to those without recurrence.
- The voltage threshold of <0.4 mV defining low voltage correlates better with the LA arrhythmogenic substrate in AT/AF patients, when both mapping catheters are considered simultaneously, than the established < 0.5 mV threshold.
- The extent of LA low voltage correlates with the one-year-risk of AT/AF recurrence.
- The one-year-risk of AT/AF recurrence increases by 3.9% for every percent of LA low voltage extent measured in the EAM.

Left atrial cardiomyopathy plays an important role in the development, maintenance, and recurrence of supraventricular arrhythmias after catheter ablation [5,9,22,23]. Not least for this reason, ablation, or the consideration of LA substrate for the ablation is coming into focus [13,15,16,21,24,25,26]. While substrate evaluation by MRI requires additional diagnostic steps, it can alternatively be assessed by bipolar low voltage as a surrogate during a 3D mapping ablation procedure [18,27,28]. But, in that regard, it is important not to equate low voltage areas in EAM with atrial cardiomyopathy. Many different factors influence the LA voltage measured in the EAM. Among others, the rhythm during the mapping plays an important role on the local bipolar voltage [29]. Especially, sinus rhythm is more reliable regarding the bipolar voltage [29], but also the count of collected electro-anatomical points plays a role in the measurement of low voltage [29]. Due to this, only anatomically complete EAM with an appropriate density of electro-anatomical points should be used for LA low voltage evaluation in EAM, as performed in this study. Furthermore, cycle length [13], myocardial wall thickness [29], electrode size and spacing [13,30], contact force [30], and wavefront orientation [31] influence the bipolar voltage measured.

The frequently used threshold value of 0.5 mV is often used for the identification of low voltage areas, which has grown historically and is mainly based on conventions [15,29,32]. Our study demonstrates that a voltage threshold of 0.4 mV has a slightly higher predictive value compared to the established 0.5 mV threshold with simultaneous consideration of both mapping catheters used in this study. Even though both mapping catheters are similar in their technical data regarding the number of electrodes, electrode size, and interelectrode distance, the two catheters differ in terms of catheter geometry. It is therefore important to note that the use of two different mapping catheters may have influenced this finding. Interestingly, Spragg et al. found in an MRI study that areas of diseased LA myocardium identified by late enhancement exhibited a voltage of 0.39 ± 0.61 mV, in contrast to heathy regions which showed a voltage of 1.38 ± 1.23 mV (*p* < 0.001) [33]. This finding supports our results.

Previous biopsy and MRI studies have shown that a significantly higher proportion of LA fibrosis in associated with a higher AT/AF recurrence [14,18].

This study demonstrates that the extent of low voltage in the LA, measured endocardially as a surrogate marker for atrial cardiomyopathy, can be used to predict AT/AF recurrence. Similar results had been shown in epicardial direct contact mapping studies [23,34,35]. Considering these studies and an endocardial study by Mitran et al., the extent of low voltage in the left atrium appears to be an independent risk factor for AT/AF recurrence [36].

LGE-MRI can be used to quantify LA cardiomyopathy. It is known that there is a strong correlation between the extent of atrial cardiomyopathy observed in MRI and the risk of AT/AF recurrence [10]. Furthermore, increased LGE values in cardiac MRI were shown to be significantly associated with lower endocardial bipolar voltages [18,33]. However, little is known about how precisely the extent of low voltage, measured in EAM, relates to the risk of recurrence. In this regard, our study demonstrates that each 1% increase in LA low voltage raises the risk of atrial tachycardia recurrence by 1.039-fold. Previous LGE-MRI and PVI studies have calculated a hazard ratio of 1.025–1.060 for recurrence per 1% increase in LA cardiomyopathy [10,15]. Our findings are consistent with these studies and reinforce the value of low voltage measurement in EAM for predicting the recurrence of AT and AF.

One cause of low voltage electrograms is atrial fibrosis [8], which can develop spontaneously or be induced iatrogenically [1,2,4,11,12], such as through ablation procedures. Intense fibrosis, which separates fibers of atrial myocardium, causes local conduction abnormalities and thus stabilizes reentry [6,37]. Furthermore, the number of AF drivers is highly dependent on the fibrosis burden, specifically in the LA, and a large proportion of these were found at the border of the fibrotic tissue [5,16]. These results can explain why patients with a larger LA low voltage extent, as a surrogate of LA cardiomyopathy, increases the risk of AT/AF recurrence in this study.

While LGE-MRI has technical advantages over voltage mapping in detecting LA cardiomyopathy, EAM may also serve as a valuable tool for identifying LA cardiomyopathy in AT/AF patients when conducted with a standardized protocol. We therefore believe that data obtained from EAM voltage maps can be used for predicting AT/AF recurrence, without the need for additional techniques like DE-MRI, which may not be widely available.

As this study demonstrates, the extent of left atrial low voltage appears to significantly influence the occurrence of supraventricular tachycardias. This parameter can be a valuable tool for explaining treatment options to patients, particularly in conveying their individual risk of developing supraventricular tachycardias after an ablation. Quantifying low voltage may help determine whether an ablation would be beneficial or if the atrium is so diseased that the risks of ablation outweigh its potential benefits. Additionally, low-voltage quantification could aid in making more objective decisions regarding the appropriateness of preventive substrate ablation. However, further prospective controlled studies are needed to validate this approach.

### Limitations

To ensure a consistent quality of mapping in this study, the cohort investigated consists of a selected population. It cannot be ruled out that this may have potentially influenced the study results.

The mapping system used in this study cannot exclusively record electroanatomical points that are in direct contact with the tissue. This limitation should be acknowledged, as the lack of electrode contact with the myocardium may lead to an underestimation of the true local voltage, potentially resulting in an overestimation of low-voltage areas. To mitigate this effect, a tissue proximity filter was applied during mapping to ensure that only electroanatomical points in close proximity to the tissue were recorded.

Furthermore, it should be noted that both prior ablations and those performed during the procedure can influence the recurrence of AT/AF and, consequently, the study results. A possible influence on the recurrence of supraventricular tachycardia by right atrial substrate was not investigated in this study, so this may also have an influence on the results of the study. 

## 5. Conclusions

According to our data, the low voltage extent in the LA is significantly associated with AT/AF recurrence within one year after catheter ablation and with the absolute post-interventional arrhythmia-free time.

## Figures and Tables

**Table 1:** Baseline characteristics of the cohort.

	All Patients n = 108	Low Voltage Extent <25% n = 53	Low Voltage Extent 25–50% n = 32	Low Voltage Extent 50–75% n = 16	Low Voltage Extent 75–100% n = 7	*p*-Value
Age [years] (mean ± SD)	68.8 ± 9.7	65.6 ± 9.8	70.0 ± 9.3	74.4 ± 7.0	75.4 ± 6.9	<0.001
Female (n [%])	44 (40.7%)	10 (18.9%)	18 (56.3%)	10 (62.5%)	6 (85.7%)	<0.001
BMI [kg/m^2^] (mean ± SD)	28.8 ± 5.0	29.0 ± 5.3	28.6 ± 4.7	28.5 ± 4.5	28.8 ± 5.4	0.982
LVEF normal (n [%]) mildly reduced (n [%]) moderately reduced (n [%]) severely reduced (n [%])	56 (51.9%) 19 (17.6%) 14 (13.0%) 19 (17.6%)	27 (50.9%) 11 (20.8%) 5 (9.4%) 10 (18.9%)	21 (65.6%) 2 (6.3%) 3 (9.4%) 6 (18.8%)	4 (25.0%) 6 (37.5%) 3 (18.8%) 3 (18.8%)	4 (57.1%) 0 (0%) 3 (42.9%) 0 (0%)	0.036
LAVi [cm^3^/m^2^] (mean ± SD)	78.7 ± 20.0	72.3 ± 18.7	83.4 ± 18.9	85.4 ± 20.9	89.1 ± 20.5	0.010
CHA_2_DS_2_-VASc (median [IQR])	3 (2–5)	3 (2–5)	3.5 (3–5)	5 (3–5.75)	5 (4–5)	0.072
Mitral valve disease † (n [%])	30 (27.8%)	12 (22.6%)	11 (34.4%)	3 (18.8%)	4 (57.1%)	0.172
preceded left atrial ablation preceded PVI (n [%]) preceded LA lines (n [%])	75 (69.4%) 5 (4.6%)	37 (69.8%) 1 (1.9%)	24 (75.0%) 3 (9.4%)	10 (62.5%) 1 (6.3%)	4 (57.1%) 0 (0%)	0.690 0.321
Preceded heart surgery (n [%])	5 (4.6%)	2 (3.8%)	1 (3.1%)	1 (6.3%)	1 (14.3%)	0.450

BMI, body mass index; IQR, interquartile range; LA, left atrial; LAVi, left atrial volume index; LVEF, left ventricular ejection fraction; PVI, pulmonary vein isolation. † mitral valve disease was defined as valve dysfunction higher than low-grade mitral-valve insufficiency or stenosis.

**Table 2:** Overview of supraventricular tachycardias treated in 108 patients.

Supraventricular Tachycardia	Count of Patients
Atrial fibrillation	96
Right atrial flutter (CTI-dependent)	6
Left atrial flutter Roof-dependent Perimitral	9 5 4
Focal atrial tachycardia Right atrial Left atrial	9 4 5

CTI, cavotricuspidal isthmus.

**Table 3:** Overview of atrial tachycardia treatment in the study population.

Supraventricular Tachycardia	Treatment	Count of Patients
Atrial fibrillation	PVI Repeat PVI substrate isolation anterior substrate isolation posterior LAA isolation	26 52 18 10 1
Right atrial flutter (CTI-dependent)	CTI isolation	6
Left atrial flutter Roof-dependent Perimitral	roof line † mitral isthmus line †	5 4
Focal atrial tachycardia Right atrial Left atrial	focal ablation focal ablation	4 5

CTI, cavotricuspidal isthmus; LAA, left atrial appendage; PVI, pulmonary vein isolation. † All flutter lines were applied based on the patient’s individual low voltage localization and extent.

**Table 4:** Logistic regression model estimating the influence of and left-atrial low voltage extent adjusted for left-atrial volume, age, sex, BMI, and relevant mitral valve disease on the one-year recurrence risk.

	Odds Ratio	95% Confidence Interval	*p*-Value
Left-atrial low voltage extent [%]	1.039	1.014–1.064	0.002
LAVi † [cm^3^/m^2^]	1.000	0.977–1.025	0.968
Age [years]	0.989	0.942–1.037	0.638
Sex	2.285	0.810–6.444	0.118
BMI [kg/m^2^]	0.949	0.863–1.043	0.275
Mitral valve disease ‡ [yes/no]	1.005	0.379–2.670	0.991

BMI, body mass index; LAVi, left atrial volume index. † Determined using electro-anatomical mapping and indexed to the body surface. ‡ Mitral valve disease was defined as valve dysfunction higher than low-grade mitral-valve-insufficiency or stenosis.

## Data Availability

The data presented in this study are available on request from the corresponding author. The data are not publicly available due to data privacy law.
